# Electrophysiological Correlates of Proactive Control and Binding Processes during Task Switching in Tourette Syndrome

**DOI:** 10.1523/ENEURO.0279-22.2023

**Published:** 2023-04-07

**Authors:** Laura Wehmeyer, Canan Beate Schüller, Theo O. J. Gruendler, Daniel Huys, Jens Kuhn, Markus Ullsperger, Veerle Visser-Vandewalle, Pablo Andrade, Juan Carlos Baldermann, Thomas Schüller

**Affiliations:** 1 Department of Stereotactic and Functional Neurosurgery, Faculty of Medicine and University Hospital Cologne, University of Cologne, Cologne 50937, Germany; 2 Department of Psychiatry and Psychotherapy, Faculty of Medicine and University Hospital Cologne, University of Cologne, Cologne 50937, Germany; 3 Center for Military Mental Health, Military Hospital Berlin, Berlin 10115, Germany; 4 Department of Psychiatry and Psychotherapy III, LVR Hospital Bonn, Bonn 53111, Germany; 5 Department of Psychiatry, Psychotherapy, and Psychosomatics, Johanniter Hospital Oberhausen, Oberhausen 46145, Germany; 6 Department of Psychology, Otto-von-Guericke University, Magdeburg 39106, Germany; 7 Center for Behavioral Brain Sciences, Magdeburg 39106, Germany; 8 Department of Neurology, Faculty of Medicine and University Hospital Cologne, University of Cologne, Cologne 50937, Germany

**Keywords:** EEG, perception-action binding, proactive control, RIDE, task switching, Tourette syndrome

## Abstract

The occurrence of tics in Tourette syndrome (TS) has often been linked to impaired cognitive control, but empirical findings are still inconclusive. A recent view proposes that tics may be the result of an abnormally strong interrelation between perceptual processes and motor actions, commonly referred to as perception-action binding. The general aim of the present study was to examine proactive control and binding effects in the context of task switching in adult human patients with TS and matched healthy controls. A cued task switching paradigm was employed in 24 patients (18 male, 6 female) and 25 controls while recording electroencephalography (EEG). Residue iteration decomposition (RIDE) was applied to analyze cue-locked proactive cognitive control and target-locked binding processes. Behavioral task switching performance was unaltered in patients with TS. A cue-locked parietal switch positivity, reflecting proactive control processes involved in the reconfiguration of the new task did not differ between groups. Importantly, target-locked fronto-central (N2) and parietal (P3) modulations, reflecting binding processes between perception and action, differed between groups. Underlying neurophysiological processes were best depicted after temporal decomposition of the EEG signal. The present results argue for unaltered proactive control but altered perception-action binding processes in the context of task switching, supporting the view that the integration of perception and action is processed differently in patients TS. Future studies should further investigate the specific conditions under which binding may be altered in TS and the influence of top-down processes, such as proactive control, on bindings.

## Significance Statement

The origin of tics in Tourette syndrome (TS) is still poorly understood. Based on the phenomenon of the premonitory urge (PMU), it has recently been proposed that tics may be the result of an abnormally strong interrelation between perceptual processes and motor actions, i.e., increased perception-action binding. In the present study, we investigated binding effects in the context of a task switching paradigm using electroencephalography (EEG) to determine underlying neurophysiological mechanisms. Our results suggest that fronto-central (N2) and parietal (P3) activity are differentially modulated by binding between perception and action in patients with Tourette syndrome, supporting the view that the integration of perception and action is processed differently and may relate to the core symptoms of the disorder, urges, and tics.

## Introduction

Tourette syndrome (TS) is a neurodevelopmental disorder characterized by motor and vocal tics, which are usually preceded by a premonitory urge (PMU) that ceases after tic execution (Brandt et al., 2016). Tics can be voluntarily suppressed for a limited period of time (Ganos et al., 2018). While the pathophysiology of TS is still incompletely understood, symptoms are assumed to be related to dysfunctions of cortico-basal ganglia-thalamo-cortical circuits with altered dopaminergic neurotransmission playing a central role (Albin and Mink, 2006; Maia and Conceição, 2018; Rae et al., 2019). The phenomenology of tics has led to the assumption that cognitive control processes might be impaired in patients with TS, but empirical findings are inconclusive (Ganos et al., 2014; Morand-Beaulieu et al., 2017). Recently, TS symptoms have been linked to an abnormally strong interrelation between perceptual processes (i.e., PMU) and motor actions (i.e., tics), commonly referred to as perception-action binding (Beste and Münchau, 2018).

**Figure 1.**
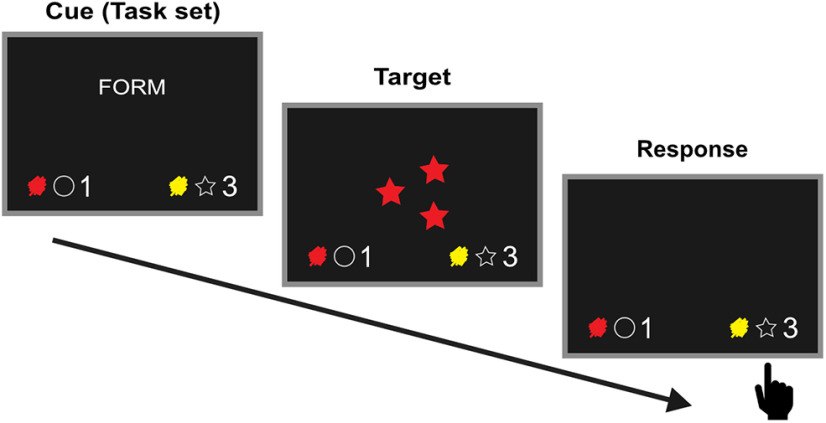
Illustration of one trial of the task switching paradigm. The cue signals which task should be performed (i.e., attend to the shape of the target stimulus), thereby activating the corresponding task set. The target stimulus then requires pressing the right key for star according to the assignment of the target features to the left or right response key in the lower corners. The timing of the stimuli is described in the text.

Both cognitive control and perception-action binding can be investigated with the cued task switching paradigm. Here, a cue signals which task should be performed (e.g., attend to the shape of the target stimulus) to select the appropriate response (e.g., pressing the right button to select the star as shown in Fig. 1). When the cue signals a switch of tasks, costs can be observed in the form of longer reaction times (RTs) and higher error rates (Monsell, 2003). Cognitive processes contributing to switch costs can be dissociated on a neurophysiological level (Jamadar et al., 2015). Proactive control processes involved in the reconfiguration of the new task set (i.e., mental representation of the task) have been associated with a sustained parietal modulation after cue onset termed “switch positivity” (Nicholson et al., 2005; Travers and West, 2008; Karayanidis and Jamadar, 2014). Of note, recent behavioral studies have shown that proactive control is likely not impaired in TS (Rawji et al., 2020; Indrajeet et al., 2022). Given sufficient time to execute proactive control, switch costs can be diminished, but residual switch costs still remain (Monsell, 2003). Residual switch costs have been attributed to interference from previous trials, which in turn is associated with target-locked frontal (N2) and parietal (P3) modulations (Allport and Wylie, 2000; Karayanidis and Jamadar, 2014; Kopp et al., 2020). Associative binding theories suggest that interference may arise from bindings between task features from the previous trial (Abrahamse et al., 2016; Frings et al., 2020). All features of the current trial including task set (activated by the cue), target and response are assumed to be stored in a common event file that may be reactivated by repetition of any feature in the following trial (Allport and Wylie, 2000; Hommel et al., 2001; Waszak et al., 2003; Koch et al., 2018). In particular, bindings between task set and target (task set-target bindings) may cause task-irrelevant target features to trigger reactivation of the previous task set (Kopp et al., 2020). Additionally, bindings between task set and response (task set-response bindings) may cause interference for switched responses on task repeat trials and repeated responses on task switch trials because of reactivation of the previous event file (Altmann, 2011; Koch et al., 2018). Recent studies particularly point to stronger associations between stimulus and response in TS, hence increased perception-action binding (Petruo et al., 2016; Kleimaker et al., 2020). Therefore, in patients with TS task set-response binding may be also altered in the context of task switching.

To date, task switching processes and corresponding electrophysiological modulations have not been investigated in TS. Therefore, our objective was to examine proactive control and binding processes in adult patients with TS and matched healthy controls using a cued task switching paradigm. Importantly, electrophysiological correlates of binding are best depicted when disentangled from pure stimulus or response processes using residue iteration decomposition (RIDE). RIDE separates the event-related potential (ERP) into a stimulus-locked S-cluster, response-locked R-cluster and intermediate C-cluster (Ouyang et al., 2011). Binding as well as task switching processes have been shown to be particularly well reflected by the C-cluster in the N2/P3 time window at both parietal and frontal electrode sites (Wolff et al., 2017; Kleimaker et al., 2020; Opitz et al., 2020).

We hypothesized unaltered behavioral task switch costs and increased task set-response binding effects in patients with TS. Furthermore, we expected a parietal switch positivity before target onset and task set-response binding modulations of frontal/parietal activity in the N2/P3 time window. We assume that these electrophysiological modulations are specific to the C-cluster rather than the S-/R-cluster or nondecomposed ERP. Finally, electrophysiological modulations were examined for group differences.

## Materials and Methods

### Participants

Twenty-five adult patients with TS were recruited at the University Hospital Cologne, and 25 healthy participants matched for gender, age, and years of education were gathered through public advertisements (for demographic data, see Table 1). One patient was excluded because of an excessive error rate (59%). Each participant was clinically assessed using standardized clinical assessments. Tic severity was scored using the clinician-rated Yale Global Tic Severity Scale (YGTSS; Leckman et al., 1989). Additional self-report scales were administered to screen for secondary and comorbid symptoms. Specifically, the PMU was measured using the Premonitory Urge for Tics Scale (PUTS; Woods et al., 2005). Obsessive-compulsive disorder (OCD) symptoms were tested with the revised Obsessive-Compulsive Inventory (OCI-R; Foa et al., 2002) and symptoms of depression were rated with the Beck Depression Inventory–Version II (BDI–II; Beck et al., 1996). Retrospective symptoms of attention-deficit hyperactivity disorder (ADHD) during childhood were scored on the Wender Utah Rating Scale (WURS-K; Retz-Junginger et al., 2002; for group comparison results, see Table 1). Of the 24 included patients, six were taking prescribed medication for the management of their tics at the time of testing. A total of five patients were treated with neuroleptics (three × aripiprazole, one × tiapride, one × risperidone) and one with tetrabenazine. These patients were asked to stop medication 24 h before the testing. All participants had normal or corrected-to-normal vision. Each participant provided oral and written informed consent. The study was approved by the Ethics Committee of the Medical Faculty of the University of Cologne (No. 16–491) and performed in accordance with the Declaration of Helsinki.

**Table 1:** Demographic data and results of group comparisons

	TS	HC	*t*	df	*p*
Age	30.21 (9.07)	29.40 (9.28)	0.308	47	0.759
Sex (M/F)	18/6	17/8	0.2941	1	0.754
Years of education	11.75 (1.22)	12.12 (1.17)	−1.083	47	0.284
BDI-II	12.13 (9.27)	5.28 (5.19)	3.207	47	0.002*
OCI-R	20.52 (12.27)	10.92 (7.58)	3.309	47	0.002*
WURS-K	26.54 (11.64)	16.04 (9.55)	3.458	47	0.001*
YGTSS total	27.63 (11.47)				
YGTSS global	53.88 (20.49)				
PUTS	30.27 (4.12)				

Data are mean (SD). TS = Tourette patients; HC = Control participants; BDI-II = Beck Depression Inventory II; OCI-R = Obsessive-compulsive Inventory Revised; WURS- K = Wender Utah Rating Scale; YGTSS = Yale Global Tic Severity Scale; PUTS = Premonitory Urge for Tics Scale. Asterisk denotes statistical significance. ^1^χ^2^.

### Experimental design

Participants performed a computer-based cued task switching paradigm (Fig. 1) which was administered using Presentation 16.3 (Neurobehavioral Systems). Responses were given via a response pad (RB-840, Cedrus). After initial practice trials, the task consisted of 432 trials. Each trial began with the presentation of one of three possible cues in German (“FARBE,” “FORM,” or “ANZAHL” corresponding to “COLOR,” “SHAPE,” or “NUMBER”) representing the task according to which the following target stimulus had to be classified. The cue was depicted for either 100 ms [short cue-target interval (CTI)] or 400 ms with a subsequent waiting interval of 400 ms (long CTI) until the target stimulus was presented for 300 ms followed by a blank screen. The target consisted of either one or three symbols, shaped as a star or circle, and colored either red or yellow. Each target feature was assigned to a left or right response key (i.e., “RED,” “CIRCLE,” and “1” = left key; “YELLOW,” “STAR,” or “3” = right key). The assignment of the features to the keys remained the same throughout the paradigm and was always displayed in the lower corners of the screen. Participants had to focus on the target feature indicated by the cue and respond as quickly as possible by pressing the appropriate key. Responses had to be executed within 1800 ms after target onset. Once the response was made or after 1800 ms, a blank screen was presented for the response-cue interval (RCI) that randomly varied between 1000, 1500, and 2000 ms before the next trial started. The number of task repeat (cue indicating the same relevant dimension as in the trial before) and task switch (cue indicating a different relevant dimension) trials was counterbalanced in a pseudo-randomized order. The task comprised of four blocks separated by short pauses, the length of which was determined by the participants.

### Electroencephalography recording and analyses

Electroencephalography (EEG) was recorded from 63 Ag/AgCl (EASYCAP GmbH) electrodes according to the extended 10–20 system. Recordings were performed with a sampling rate of 5000 Hz and all impedances were kept below 15 kΩ. Data were preprocessed and analyzed offline using EEGLAB 2022.1 (Delorme and Makeig, 2004) and custom MATLAB R2021b routines (The MathWorks). The data were filtered using a finite impulse response filter with cutoff frequencies of 0.5 and 40 Hz (6 dB/octave) and resampled to 500 Hz. Abnormal channels with a low correlation with neighboring channels (channel criterion = 0.8) were removed (removed channels: TS: 1.21 ± 2.11 SD, Controls: 1.64 ± 1.73 SD) and interpolated using spherical splines (Perrin et al., 1989). Between-block rest periods and redundant data before and after the task were also removed. Then, EEG data were re-referenced to an average reference and the FCz reference channel was added back. For the identification of artifacts, an extended infomax independent component analysis (ICA) was run on the continuous data. Resulting independent components were then submitted to the fully automated artifact classifier MARA (Winkler et al., 2011). A total of 18.75 ± 7.01 (SD) independent components remained for the TS group and 25.00 ± 9.07 (SD) for the control group. Next, cue-locked epochs were created from 500 ms before to 3000 ms after cue onset and baseline-corrected by removing the mean voltage calculated over the time window of 200 ms before cue onset. For each participant, the first trial in each block was removed. Additionally, only correct trials that also followed a correct trial with a CTI of 800 ms and with reaction times (RTs) below 1800 ms were considered for further analysis steps.

For the cue-locked analysis, subepochs from 200 ms before to 1000 ms after cue onset were extracted and divided into separate segments for each Task Transition condition (i.e., task repeat, task switch). An automated artifact rejection based on extreme values and improbability was applied to the segmented data (Delorme et al., 2007). Epochs were rejected if amplitudes reached a threshold of ± 150 μV or the joint data probability exceeded 5 standard deviations (average rejected epochs per condition: TS: 3.25 ± 1.64 SD, Controls: 3.44 ± 1.38 SD). Further, current source density (CSD) transformation was performed using the potential difference between one electrode and the potential total of all surrounding electrodes (Kayser and Tenke, 2006). To perform traditional ERP analyses, trials were averaged for each condition and subject, and mean amplitudes were extracted for the switch positivity at left lateral parieto-occipital electrodes (P5/PO3/PO7) over a time window of 400–800 ms after cue onset. The choice of electrodes was confirmed by a validation method in which the differential mean activity (task switch – task repeat) of each electrode was compared with that of all other electrodes using false discovery rate (FDR) for multiple comparison correction (adjusted threshold of *p *<* *0.0007).

For the target-locked analysis, target-locked epochs from −200 to 2100 ms (the upper epoch limit ensures that the epoch time window covers up to 300 ms after the latest possible response) were generated. Separate segments were created for each Task Transition/Response Transition condition (i.e., task repeat and response repeat, task repeat and response switch, task switch and response repeat, task switch and response switch). Following the same procedure as for the cue-locked analysis, artifactual epochs were rejected (average rejected epochs per condition: TS: 1.16 ± 0.84 SD, Controls: 1.28 ± 1.16 SD) and CSD transformation was applied. After averaging the ERP over trials for each condition and subject, mean amplitudes were calculated within a time window of 200−500 ms after target onset. Fronto-central electrode Cz was selected for the N2 component and left lateral parieto-occipital electrodes (P5/PO3/PO7) for the P3 component. Similar to the cue-locked analysis, the choice of electrodes was confirmed by the same validation method, but this time using mean amplitudes.

In a next step, the segmented single-trial data were temporally decomposed using the RIDE toolbox (for further details, see http://cns.hkbu.edu.hk/RIDE.htm; Ouyang et al., 2015a,b). For the cue-locked data, RIDE clusters were derived from a prespecified time window from 0 to 600 ms after cue onset for the S-cluster and from 200 to 800 ms for the C-cluster. For the target-locked data, the following RIDE clusters were extracted: S-cluster from 0 to 600 ms after target onset, the C-cluster from 150 to 1000 ms, and the R-cluster from −300 to 300 ms around the response. To quantify the mean amplitudes in each of the obtained RIDE clusters, we focused on the same time windows and electrodes as described above for the cue-locked and target-locked ERP analyses. Also, the same validation methods were used to confirm electrode sites and time windows for the C-cluster.

### Statistical analysis

Statistical analyses were performed with SPSS 29 (IBM Corp.). For analyses of the behavioral data, repeated-measures ANOVAs with “Group” (patients, controls) as between-subject factor and “Task Transition” (repeated vs switched task) and “Response Transition” (repeated vs switched response) as within-subject factors were performed for RTs and error rates. In correspondence with the EEG analyses, only correct trials that also followed a correct trial, with a CTI of 800 ms and with RTs below 1800 ms were included. The first trial in each block was excluded. For analyses of the neurophysiological data, mean amplitudes of each RIDE cluster and the standard ERP were analyzed using repeated-measures ANOVAs with “Group” as between-subject factor and “Task Transition” and “Response Transition” as within-subject factors. However, the latter within-subject factor was only included for the target-locked analysis. Significant ANOVA effects were followed up with Bonferroni-corrected *post hoc* pairwise comparisons. In addition, a repeated-measures ANCOVA with “Medication” (nonmedicated vs medicated) as covariate was performed to control for medication as a confounding factor. Effect sizes are reported as partial η^2^ ( ${\text{ηρ}}^{2}$). The Bayesian posterior probability of the null hypothesis being true given the observed data (*p*_BIC_(H_0_|D)) is reported when of theoretical importance. In doing so, we followed the method proposed by Masson (2011) based on Wagenmakers (2007), which generates Bayesian probabilities using the Bayesian information criterion (BIC) estimate of the Bayes factor derived from ANOVA sum of squares. Obtained probabilities are interpreted according to the classification scheme of Raftery (1995; i.e., 0.50–0.75 = weak evidence; 0.75–0.95 = positive evidence; 0.95–0.99 = strong evidence; > 0.99 = very strong evidence). In an exploratory analysis, Spearman’s correlations were calculated to test the relationship between behavioral and neurophysiological effects and clinical parameters (YGTSS total tic, PUTS, BDI-II, OCI-R, WURS-K) for the TS group only.

## Results

### Behavior

**Figure 2.**
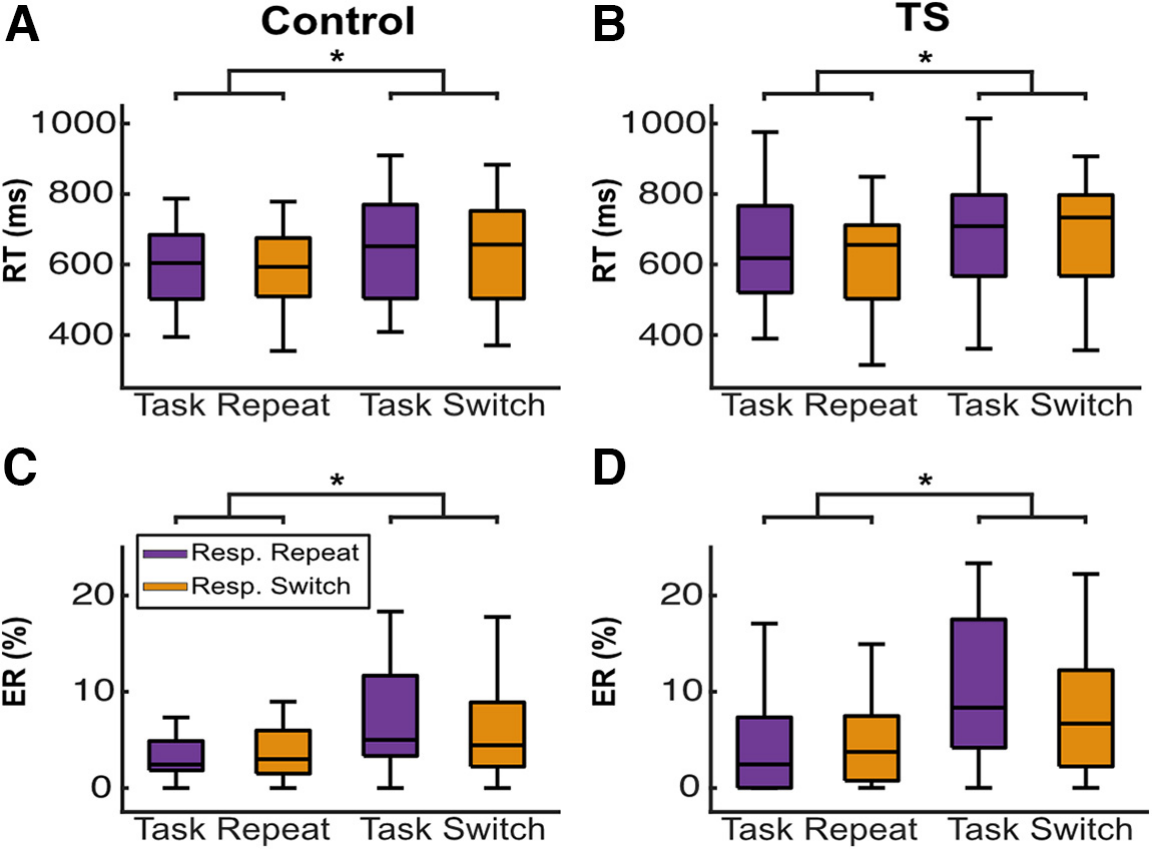
Behavioral results. Boxplots for reaction times (RTs) and error rates (ERs) separately for controls and patients. ***A***, RTs for controls. ***B***, RTs for patients. ***C***, ERs for controls. ***D***, ERs for patients. Asterisks denote significant differences between experimental conditions. Resp. = Response.

The RTs and error rates of each group are shown in Figure 2. For RTs, there was a significant main effect of Task Transition (*F*_(1,47)_ = 45.71, *p *<* *0.001, ${\text{ηρ}}^{2}$ = 0.493) with slower RTs on task switch than task repeat trials, which might indicate proactive control and/or task set-target binding processes. No main effects of Response Transition (*F*_(1,47)_ = 2.63, *p *=* *0.111, ${\text{ηρ}}^{2}$ = 0.053) or Group (*F*_(1,47)_ = 1.30, *p *=* *0.260, ${\text{ηρ}}^{2}$ = 0.027) were observed. The interactions between Task Transition × Group (*F*_(1,47)_ = 0.77, *p *=* *0.386, ${\text{ηρ}}^{2}$ = 0.016) and Response Transition × Group (*F*_(1,47)_ = 1.02, *p *=* *0.318, ${\text{ηρ}}^{2}$ = 0.021) were also nonsignificant. Importantly, task set-response binding processes would be indicated by an interaction between Task Transition and Response Transition. However, both interaction effects between Task Transition × Response Transition (*F*_(1,47)_ = 0.02, *p *=* *0.896, ${\text{ηρ}}^{2}$ = 0.000, *p*_BIC_(H_0_|D) = 0.874) and Task Transition × Response Transition × Group (*F*_(1,47)_ = 0.12, *p *=* *0.735, ${\text{ηρ}}^{2}$ = 0.002, *p*_BIC_(H_0_|D) = 0.868) were nonsignificant, with Bayesian analyses providing positive evidence for the null hypotheses. After including medication as a covariate, the Task Transition main effect remained significant (*F*_(1,46)_ = 32.43, *p *<* *0.001, ${\text{ηρ}}^{2}$ = 0.413). The analysis of error rates revealed a significant main effect of Task Transition (*F*_(1,47)_ = 53.33, *p *< 0.001, ${\text{ηρ}}^{2}$ = 0.532), suggesting that error rates increased for task switch trials. Again, no main effect of Response Transition (*F*_(1,47)_ = 1.48, *p *=* *0.229, ${\text{ηρ}}^{2}$ = 0.031) or Group (*F*_(1,47)_ = 1.90, *p *=* *0.174, ${\text{ηρ}}^{2}$ = 0.039) was found, and no interactions between Task Transition × Group (*F*_(1,47)_ = 1.93, *p *=* *0.171, ${\text{ηρ}}^{2}$ = 0.040) and Response Transition × group (*F*_(1,47)_ = 0.93, *p *=* *0.341, ${\text{ηρ}}^{2}$ = 0.019). Similarly, the interactions between Task Transition × Response Transition (*F*_(1,47)_ = 2.85, *p *=* *0.098, ${\text{ηρ}}^{2}$ = 0.057) and Task Transition × Response Transition × Group (*F*_(1,47)_ = 0.17, *p *=* *0.685, ${\text{ηρ}}^{2}$ = 0.004) were not significant. However, Bayesian analyses provided only weak evidence for the null hypothesis of the former (*p*_BIC_(H_0_|D) = 0.624), but positive evidence for the latter (*p*_BIC_(H_0_|D) = 0.865). When controlling for medication, the Task Transition main effect remained significant (*F*_(1,46)_ = 43.39, *p *<* *0.001, ${\text{ηρ}}^{2}$ = 0.485).

### Neurophysiology

#### Cue-locked parietal switch positivity

**Figure 3.**
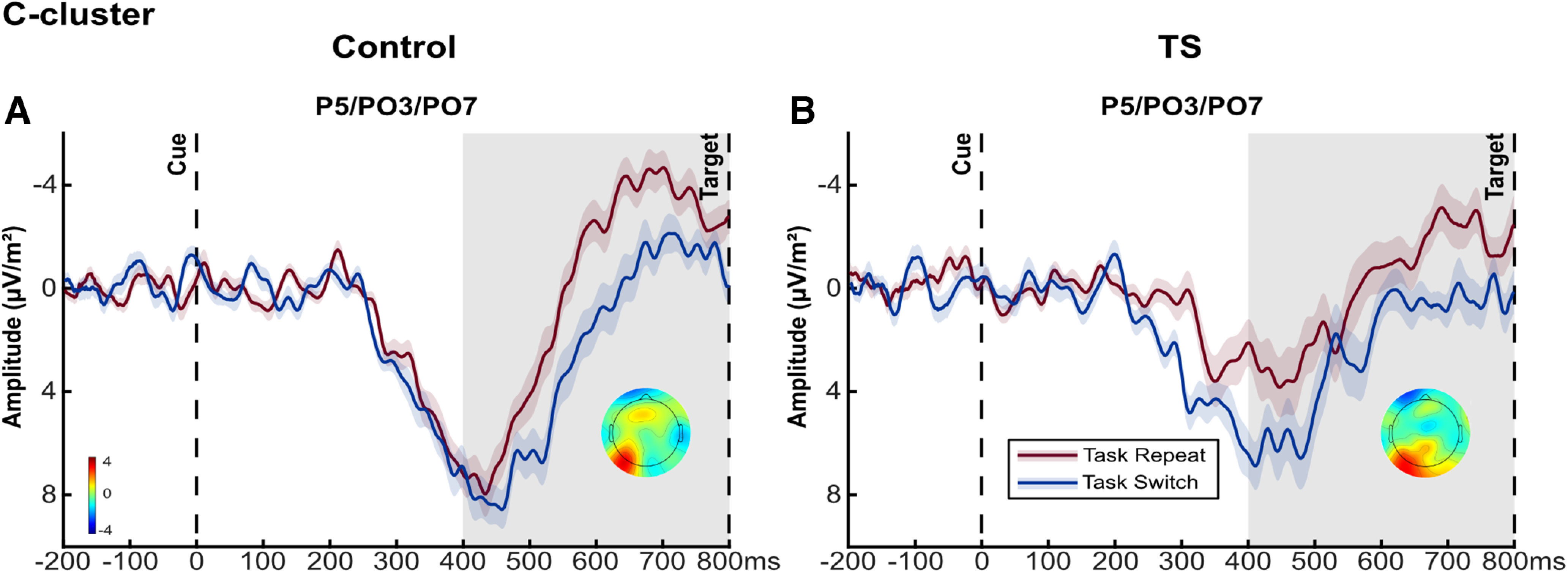
Cue-locked switch positivity results. Grand average cue-locked waveforms at electrodes P5/PO3/PO7 in the C-cluster separately for controls and patients (***A***) in the control group and (***B***) in the TS group. Shading represents standard errors (SE). The gray bar indicates the time window for mean amplitude quantification (400–800 ms). Scalp topography maps show the differences in mean amplitude (task switch – task repeat) in the respective time window. See Extended Data Figure 3-1 for the ANOVA results in the S-cluster and ERP and Extended Data Figure 3-2 for the corresponding waveforms.

In the C-cluster, cue-locked parietal activity was significantly modulated by Task Transition (*F*_(1,47)_ = 31.12, *p *<* *0.001, ${\text{ηρ}}^{2}$ = 0.398), corresponding to an increased positivity for task switch trials (Fig. 3). The Group main effect (*F*_(1,47)_ = 0.00, *p *=* *0.998, ${\text{ηρ}}^{2}$ = 0.000) and Task Transition × Group interaction effect (*F*_(1,47)_ = 0.00, *p *=* *0.948, ${\text{ηρ}}^{2}$ = 0.000) were nonsignificant. Bayesian analysis provided positive evidence for a similar effect of Task Transition in both groups (*p*_BIC_(H_0_|D) = 0.875). The Task Transition main effect was not modified by medication (*F*_(1,46)_ = 26.23, *p *<* *0.001, ${\text{ηρ}}^{2}$ = 0.363).

10.1523/ENEURO.0279-22.2023.f3-1 Extended Data Figure 3-1 Cue-locked switch positivity ANOVA results for the S-cluster and ERP. Significant ANOVA effects were followed up by an ANCOVA with Medication as covariate. Asterisk denotes statistical significance. P(H_0_|D) *=* probability of the null hypothesis being true given the observed data. See Extended Data Figure 3-2 for the corresponding waveforms. Download Figure 3-1, DOC file.

10.1523/ENEURO.0279-22.2023.f3-2 Extended Data Figure 3-2 Cue-locked switch positivity results. Grand average cue-locked waveforms at electrodes P5/PO3/PO7 in the S-cluster and standard ERP separately for controls and patients. ***A***, S-cluster waveform in the control group. ***B***, S-cluster waveform in the TS group. ***C***, ERP waveform in the control group. ***D***, ERP waveform in the TS group. Shading represents SE. The grey bar indicates the time window for mean amplitude quantification (400–800 ms). Scalp topography maps show the differences in mean amplitude (task switch–task repeat) in the respective time window. See Extended Data Figure 3-1 for the corresponding ANOVA results. Download Figure 3-2, TIF file.

In the S-cluster and conventional ERP, similar effects were observed (Extended Data Figs. 3-1, 3-2).

#### Target-locked frontal N2

**Figure 4.**
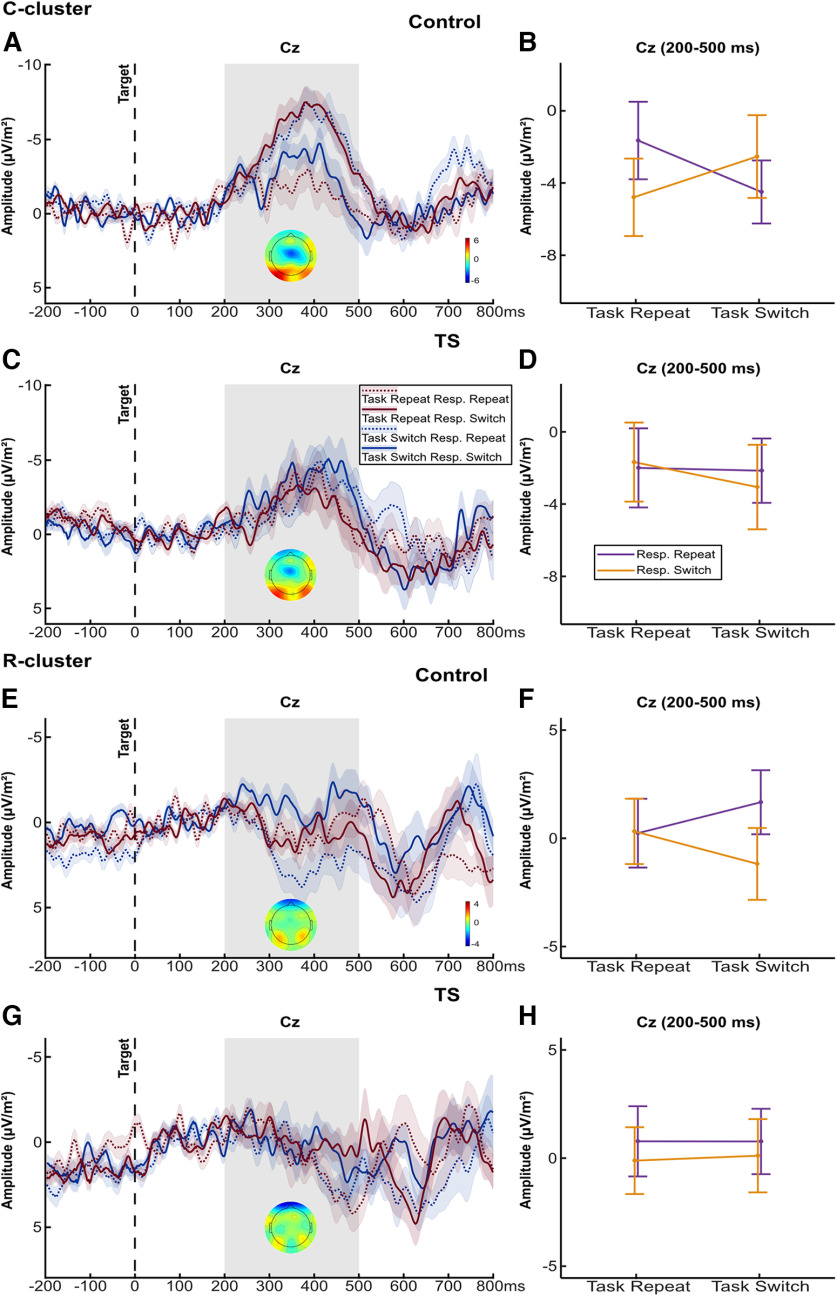
Target-locked N2 results. Grand average target-locked waveforms at electrode Cz in the C- and R-cluster separately for controls and patients. ***A***, C-cluster waveform at Cz in the control group. ***B***, C-cluster mean amplitudes at Cz in the time interval 200–500 ms in the control group. ***C***, C-cluster waveform in the TS group. ***D***, C-cluster mean amplitudes in the TS group. ***E***, R-cluster waveform in the control group. ***F***, R-cluster mean amplitudes in the control group. ***G***, R-cluster waveform in the TS group. ***H***, R-cluster mean amplitudes in the TS group. Shading and error bars indicate standard errors (SE). The gray bar indicates the time window for mean amplitude quantification (200–500 ms). Scalp topography maps show mean amplitudes in the respective time window. Resp. = Response. See Extended Data Figure 4-1 for the ANOVA results in the S-cluster and ERP and Extended Data Figure 4-2 for the corresponding waveforms.

In the C-cluster, no main effects of Task Transition (*F*_(1,47)_ = 0.84, *p *=* *0.364, ${\text{ηρ}}^{2}$ = 0.018), Response Transition (*F*_(1,47)_ = 0.56, *p *=* *0.459, ${\text{ηρ}}^{2}$ = 0.012), or Group (*F*_(1,47)_ = 1.02, *p *=* *0.317, ${\text{ηρ}}^{2}$ = 0.021) were observed. Similarly, there were no significant two-way interactions (all *p *>* *0.064). However, the three-way interaction between Task Transition × Response Transition × Group was significant (*F*_(1,47)_ = 9.60, *p *=* *0.003, ${\text{ηρ}}^{2}$ = 0.170). Importantly, this three-way interaction points to differential task set-response binding processes between groups. In the control group, *post hoc* pairwise comparisons demonstrated a significantly increased negativity for switched responses compared with repeated responses on task repeat trials (*p *=* *0.004), and a numerically increased negativity for repeated responses compared with switched responses on task switch trials, but this effect was nonsignificant (*p *=* *0.097; Fig. 4*A*,*B*). In the TS group, however, none of the *post hoc* pairwise contrasts were significant (all *p *>* *0.448; Fig. 4*C*,*D*). Importantly, the three-way interaction remained significant when controlling for medication (*F*_(1,46)_ = 10.65, *p *=* *0.002, ${\text{ηρ}}^{2}$ = 0.188).

10.1523/ENEURO.0279-22.2023.f4-1 Extended Data Figure 4-1 Target-locked N2 ANOVA results for the S-cluster and ERP. Significant ANOVA effects were followed up by an ANCOVA with Medication as covariate. Asterisk denotes statistical significance. P(H_0_|D) *=* probability of the null hypothesis being true given the observed data. See Extended Data Figure 4-2 for the corresponding waveforms. Download Figure 4-1, DOC file.

10.1523/ENEURO.0279-22.2023.f4-2 Extended Data Figure 4-2 Target-locked N2 results. Grand average target-locked waveforms at electrode Cz in the S-cluster and standard ERP separately for controls and patients. ***A***, S-cluster waveform in the control group. ***B***, S-cluster waveform in the TS group. ***C***, ERP waveform in the control group. ***D***, ERP waveform in the TS group. Shading represents SE. The grey bar indicates the time window for mean amplitude quantification (200−500 ms). Scalp topography maps show mean amplitudes in the respective time window. Resp. = Response. See Extended Data Figure 4-1 for the corresponding ANOVA results. Download Figure 4-2, TIF file.

In the R-cluster, a significant main effect of Response Transition (*F*_(1,47)_ = 7.72, *p *=* *0.008, ${\text{ηρ}}^{2}$ = 0.141) was observed, indicating an increased positivity for repeated response compared with switched responses. There were no significant main effects of Task Transition (*F*_(1,47)_ = 0.01, *p *=* *0.935, ${\text{ηρ}}^{2}$ = 0.001) or Group (*F*_(1,47)_ = 0.02, *p *=* *0.885, ${\text{ηρ}}^{2}$ = 0.000). Also, no significant two-way interactions were found (all *p *>* *0.071). Importantly, similar to the C-cluster, the three-way interaction between Task Transition × Response Transition × Group was significant (*F*_(1,47)_ = 4.68, *p *=* *0.036, ${\text{ηρ}}^{2}$ = 0.090). In the control group, *post hoc* pairwise comparisons demonstrated a significantly increased positivity for repeated responses compared with switched responses on task switch trials (*p *<* *0.001), while Response Transition conditions did not differ on task repeat trials (*p *=* *0.910; Fig. 4*E*,*F*). In the TS group, none of the *post hoc* pairwise contrasts were significant (all *p *>* *0.229; Fig. 4*G*,*H*). When medication was included as covariate, the effects of Response Transition and Task Transition × Response Transition × Group remained significant (*F*_(1,46)_ = 4.15, *p *=* *0.047, ${\text{ηρ}}^{2}$ = 0.083; *F*_(1,46)_ = 4.73, *p *=* *0.035, ${\text{ηρ}}^{2}$ = 0.083, respectively).

In the S-cluster and standard ERP, no significant effects were found after controlling for medication (Extended Data Figs. 4-1, 4-2).

#### Target-locked parietal P3

**Figure 5.**
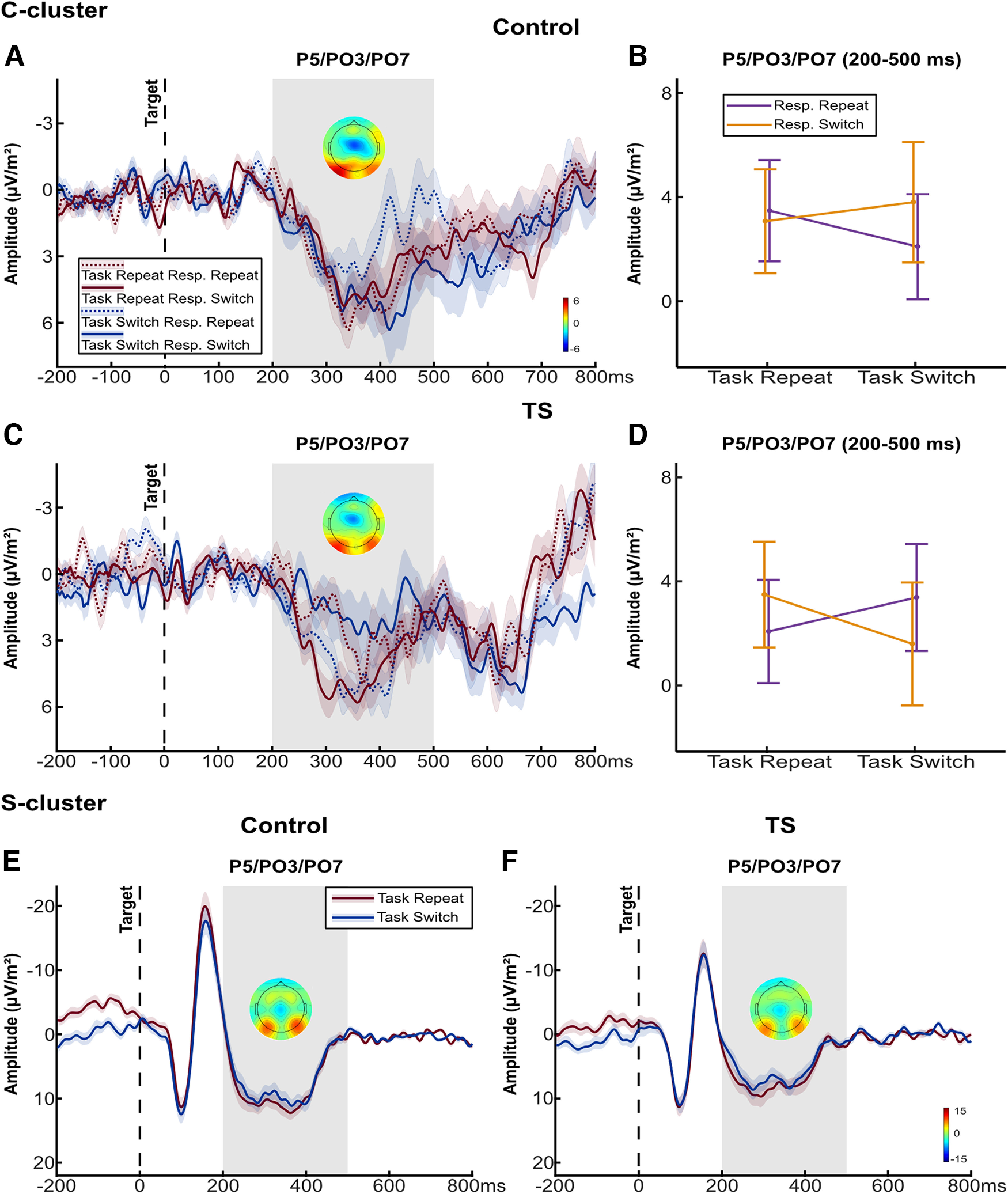
Target-locked P3 results. Grand average target-locked waveforms at electrodes P5/PO3/PO7 in the C- and S-cluster separately for controls and patients. ***A***, C-cluster waveform at P5/PO3/PO7 in the control group. ***B***, C-cluster mean amplitudes at P5/PO3/PO7 in the time interval 200−500 ms in the control group. ***C***, C-cluster waveform in the TS group. ***D***, C-cluster mean amplitudes in the TS group. ***E***, S-cluster waveform in the control group. ***F***, S-cluster waveform in the TS group. Shading and error bars indicate standard errors (SE). The gray bar indicates the time window for mean amplitude quantification (200–500 ms). Scalp topography maps show mean amplitudes in the respective time window. Resp. = Response. See Extended Data Figure 5-1 for the ANOVA results in the R-cluster and ERP, and Extended Data Figure 5-2 for the corresponding R-cluster/ERP waveforms, as well as the S-cluster waveform for each experimental condition.

In the C-cluster, there were no significant main effects of Task Transition (*F*_(1,47)_ = 0.44, *p *=* *0.511, ${\text{ηρ}}^{2}$ = 0.009), Response Transition (*F*_(1,47)_ = 2.15, *p *=* *0.645, ${\text{ηρ}}^{2}$ = 0.058), or Group (*F*_(1,47)_ = 0.15, *p *=* *0.697, ${\text{ηρ}}^{2}$ = 0.003). Similarly, no significant two-way interactions were found (all *p *>* *0.408). However, a significant three-way interaction between Task Transition × Response Transition × Group was observed (*F*_(1,47)_ = 7.48, *p *=* *0.009, ${\text{ηρ}}^{2}$ = 0.137), indicating differential task set-response binding processes between groups. In the control group, *post hoc* analysis revealed a significantly increased positivity for switched responses compared with repeated responses on task switch trials (*p *=* *0.040), whereas Response Transition conditions did not differ significantly on task repeat trials (*p *=* *0.719; Fig. 5*A*,*B*). In the TS group, the opposite pattern was observed: a significantly increased positivity for repeated responses compared with switched responses on task switch trials (*p *=* *0.035), whereas the effect of Response Transition on task repeat trials was also not significant (*p *=* *0.222; Fig. 5*C*,*D*). When accounting for a potentially confounding effect of medication, the three-way interaction remained significant (*F*_(1,46)_ = 6.40, *p *=* *0.015, ${\text{ηρ}}^{2}$ = 0.122).

10.1523/ENEURO.0279-22.2023.f5-1 Extended Data Figure 5-1 Target-locked P3 ANOVA results for the R-cluster and ERP. Significant ANOVA effects were followed up by an ANCOVA with Medication as covariate. Asterisk denotes statistical significance. P(H_0_|D) *=* probability of the null hypothesis being true given the observed data. See Extended Data Figure 5-2 for the corresponding waveforms. Download Figure 5-1, DOC file.

10.1523/ENEURO.0279-22.2023.f5-2 Extended Data Figure 5-2 Target-locked P3 results. Grand average target-locked waveforms at electrodes P5/PO3/PO7 in the S-cluster, R-cluster, and standard ERP separately for controls and patients. ***A***, S-cluster waveform in the control group. ***B***, S-cluster waveform in the TS group. ***C***, R-cluster waveform in the control group. ***D***, R-cluster waveform in the TS group. ***E***, ERP waveform in the control group. ***F***, ERP waveform in the TS group. Shading represents SE. The grey bar indicates the time window for mean amplitude quantification (200−500 ms). Scalp topography maps show mean amplitudes in the respective time window. Resp. = Response. See Extended Data Figure 5-1 for the corresponding ANOVA results for the R-cluster and ERP. Download Figure 5-2, TIF file.

In the S-cluster, mean amplitudes of the target-locked parietal P3 were significantly modulated by Task Transition (*F*_(1,47)_ = 6.68, *p *=* *0.013, ${\text{ηρ}}^{2}$ = 0.124), indicating an increased positivity for task repeat compared with task switch trials (Fig. 5*E*,*F*). The main effects of Response Transition (*F*_(1,47)_ = 1.08, *p *=* *0.304, ${\text{ηρ}}^{2}$ = 0.023) and Group (*F*_(1,47)_ = 0.94, *p *=* *0.336, ${\text{ηρ}}^{2}$ = 0.020) were not significant, including the two-way interactions between Task Transition × Group (*F*_(1,47)_ = 0.12, *p *=* *0.726, ${\text{ηρ}}^{2}$ = 0.003), Response Transition × Group (*F*_(1,47)_ = 0.66, *p *=* *0.423, ${\text{ηρ}}^{2}$ = 0.014), and Task Transition × Response Transition (*F*_(1,47)_ = 1.81, *p *=* *0.185, ${\text{ηρ}}^{2}$ = 0.037). Additionally, Bayesian analysis provided positive evidence for a similar effect of Task Transition in both groups (*p*_BIC_(H_0_|D) = 0.868). The three-way interaction between Task Transition × Response Transition × Group was also nonsignificant (*F*_(1,47)_ = 0.09, *p *=* *0.764, ${\text{ηρ}}^{2}$ = 0.002, *p_BIC_*(H_0_|D) = 0.870), which was supported by Bayesian analysis yielding positive evidence for the null hypothesis (Extended Data Fig. 5-2*A*,*B*). The main Task Transition effect was not influenced by medication (*F*_(1,46)_ = 11.66, *p *=* *0.001, ${\text{ηρ}}^{2}$ = 0.202).

While no significant effects were found in the R-cluster, mean amplitudes in the ERP were similarly modulated as in the S-cluster (Extended Data Figs. 5-1, 5-2*C–F*).

### Exploratory correlations

For the behavioral effects, task switch costs (task switch-RT – task repeat-RT, task switch-ER – task repeat-ER) were computed and correlated with clinical scores in the TS group. No significant correlations were observed (all *r *<* *0.30; *p *>* *0.161; see Table 2). For the neurophysiological effects, differential mean activity between conditions corresponding to neurophysiological findings in the TS group (i.e., C-cluster switch positivity: task switch – task repeat, R-cluster N2: response repeat – response switch, C-cluster P3: response repeat – response switch in task switch, S-cluster P3: task repeat – task switch) was quantified and correlated with clinical measurements. We found an uncorrected negative correlation between the parietal S-cluster Task Transition effect and PUTS scores (*r* = −0.48; *p *=* *0.019), indicating smaller Task Transition effects with increased PMU severity. No further significant correlations between other neurophysiological effects and clinical scores (all *r *<* *0.34; *p *>* *0.113) were observed (see Table 2).

**Table 2:** Spearman’s correlation coefficients

		YGTSS total	PUTS	BDI-II	OCI-R	WURS-K
RT Task Transition (task switch – task repeat)	*r*	0.20	−0.08	0.24	−0.14	0.30
	*p*	0.378	0.700	0.254	0.507	0.161
ER Task Transition (task switch – task repeat)	*r*	−0.04	−0.24	0.15	−0.13	−0.17
	*p*	0.853	0.260	0.481	0.555	0.427
C-SP Task Transition (task switch – task repeat)	*r*	−0.33	0.24	−0.05	0.06	0.30
	*p*	0.121	0.265	0.823	0.765	0.157
R-N2 Response Transition (response repeat – response switch)	*r*	0.32	−0.09	0.157	0.166	0.20
	*p*	0.125	0.669	0.465	0.437	0.361
C-P3 Response Transition (response repeat – response switch) in Task Switch	*r*	−0.14	0.201	−0.11	0.06	−0.17
	*p*	0.947	0.346	0.466	0.785	0.438
S-P3 Task Transition (task repeat – task switch)	*r*	−0.33	−0.48	−0.02	−0.19	−0.30
	*p*	0.113	0.019*	0.938	0.386	0.149

RT = Reaction time; ER = Error rate; C-SP = C-cluster switch positivity; R-N2 = R-cluster N2; C-P3 = C-cluster P3; S-P3 = S-cluster P3; YGTSS = Yale Global Tic Severity Scale; PUTS = Premonitory Urge for Tics Scale; BDI-II = Beck Depression Inventory II; OCI-R = Obsessive-compulsive Inventory Revised; WURS- K = Wender Utah Rating Scale. Asterisk denotes statistical significance.

## Discussion

In the present study, we examined cue-locked proactive control and target-locked binding processes during task switching in adult patients with TS and matched healthy controls using residue iteration decomposition (RIDE).

The present results indicate that both groups showed the expected behavioral switch costs (i.e., increased reaction times and error rates on task switch trials), which are generally attributed to proactive control processes for task-set reconfiguration as well as to interference caused by bindings between task set and target features of the previous trial (Monsell, 2003; Abrahamse et al., 2016). As hypothesized, task switch costs did not differ between groups, and no relationship between tic-severity and switch costs was found, likely indicating that proactive control is unaltered in TS in line with former behavioral studies (Morand-Beaulieu et al., 2017; Rawji et al., 2020; Indrajeet et al., 2022). This was further corroborated by our neurophysiological results, where both groups exhibited a similar cue-locked switch positivity, which has been suggested to reflect proactive reconfiguration of the new task set (Kieffaber and Hetrick, 2005; Nicholson et al., 2005; Rushworth et al., 2005; Lavric et al., 2008; Travers and West, 2008; Karayanidis and Jamadar, 2014). Additionally, the comparable behavioral task switch costs between groups indicate unaltered task set-target binding in TS. This is consistent with predictions from perception-action binding accounts, which state that altered binding in TS specifically includes actions (Beste and Münchau, 2018). In support of this, both groups showed similarly modulated activity in the S-cluster that strongly resembles the previously reported task switch P3, which presumedly represents processes necessary to overcome target-driven interference induced by task set-target bindings (Kieffaber and Hetrick, 2005; Karayanidis and Jamadar, 2014; Jamadar et al., 2015). The P3 modulation was most pronounced in the S-cluster, which is thought to primarily reflect stimulus processes, i.e., perception and attention (Ouyang et al., 2011). Interestingly, we found an, albeit exploratory, negative relationship between the S-cluster P3 and PMU severity, indicating a smaller difference between task conditions with increased urge severity. This might indicate disrupted representations of task set-target bindings or diminished activation of processes involved in overcoming task set-target binding-induced interference in patients that experience severe urges, either way suggesting a association between altered perceptual binding processes and PMU.

Generally, task set-response bindings are represented behaviorally by costs (i.e., increased reaction times and error rates) for switched responses on task repeat trials and for repeated responses on task switch trials (Gade et al., 2014; Koch et al., 2018). Contrary to our expectations, we did not observe such a behavioral effect in either group. This implies that the binding between task set and response, or the retrieval (reactivation) of this binding, was not strong enough to impact behavior (Dutzi and Hommel, 2009; Frings et al., 2020; Hommel, 2022). When comparing our study to others investigating perception-action binding, it is important to keep in mind that our task notably differs from the commonly used visual-motor event file task (Colzato et al., 2006). In particular, we examined bindings between task set and response and not between target and response. Task set-response bindings can be modulated by proactive control processes between cue and target, which is not the case for target-response bindings. It can be speculated that proactive control processes promoting the current goal (i.e., task set) may have influenced the likelihood of binding or retrieval of bindings, which may have contributed to the fact that participants did not show task set-response binding effects on a behavioral level as expected (Dutzi and Hommel, 2009; Hommel, 2022). Nevertheless, our decomposed EEG data provide evidence that target-locked processes in fronto-central (N2) as well as parietal (P3) regions were indeed influenced by task set-response (perception-action) binding.

First, we observed task set-response binding in the C-cluster N2, which is in line with previous findings showing that C-cluster activation reflects perception-action binding particularly well (Kleimaker et al., 2020; Opitz et al., 2020). Importantly, C-cluster N2 modulation related to task set-response binding was observed in the control group only, whereas there was no such modulation in the TS group. Increased N2 amplitudes have been consistently linked to increasing levels of interference and have been suggested to play an important role in conflict resolution and response selection (Karayanidis et al., 2003; Gajewski et al., 2010; Karayanidis and Jamadar, 2014). A recent study also demonstrated that fronto-central C-cluster activity was similarly modulated by distractor-response binding (Opitz et al., 2020). Based on this, we speculate that the C-cluster N2 effect in the control group represents a process of conflict resolution and is thus related to overcoming task set-response binding-induced interference. However, because this modulation is not corroborated by a corresponding behavioral task set-response binding effect, no conclusion can be drawn regarding the behavioral significance of this modulation. Nevertheless, the lack of fronto-central modulation in the TS group suggests binding-induced conflict is processed differently. Of note, R-cluster N2 activity was also modulated by task set-response binding, albeit with a smaller effect size than in the C-cluster. This modulation is unlikely to represent binding processes per se, but may be related to pure motor processes in line with the conceptualized role of R-cluster activations (Ouyang et al., 2011). Additionally, the parietal C-cluster P3 modulation by task set-response binding also differed between groups. While C-cluster P3 activation decreased for repeated responses on task switch trials in the control group, it increased in the TS group. The observed C-cluster P3 modulations in the control group are in line with findings showing that the task switching N2 and P3 are tightly coupled and increased N2 amplitudes are consistently accompanied by decreased P3 amplitudes (Karayanidis and Jamadar, 2014). Also, decreased C-cluster P3 activation has been repeatedly linked to increased perception-action binding-induced interference when response selection became more difficult and rebinding processes more complex (Petruo et al., 2016; Kleimaker et al., 2020; Takacs et al., 2020). Again, we want to point out that our findings are not corroborated by corresponding behavioral effects and therefore we can only speculate that this modulation is likewise related to binding-induced interference requiring a rebinding. Interestingly, our results show that the C-cluster P3 is oppositely modulated in the TS group, with increased activation in response to binding-induced interference. A recent study investigating perception-action binding in TS reported a similar finding, with parietal C-cluster amplitudes increasing in the less compatible condition (Kleimaker et al., 2020). Although the underlying process behind this modulation in the TS group is currently unclear, our results corroborate that parietal processes related to perception-action binding are altered in patients with TS.

The present findings complement the existing literature by demonstrating that proactive control processes in the context of task switching are not impaired in patients with TS. Rather, our neurophysiological results support the recent view that perception-action binding is altered in TS. Our results highlight that, above all, the interrelation of sensory and motor processes is highly relevant for a better understanding of the complex symptomatology of TS. This especially relates to the relationship between urges and tics, implying that the investigation of solely sensory processes (PMU) or motor actions (tics) are likely insufficient for this purpose.

Several limitations of the present study need to be addressed. First, the sample size is rather small, which limits statistical power. Second, effects of target-response bindings could not be examined in the present study because the trial structure of the paradigm was not counterbalanced to allow for a reliable assessment. Third, five patients were taking neuroleptic medications regularly but paused 24 h before testing to minimize acute effects. However, an effect of medication cannot be completely ruled out. To account for a potentially confounding effect, we report medication as a covariate. Fourth, patients with TS showed significant elevated scores on depression, ADHD, and OCD questionnaires, which could have influenced our results. However, comorbidity scores did not correlate with task modulations. Last, we would like to emphasize that neurophysiological correlates of task set-response binding did not have a decisive influence on behavioral performance. Therefore, it is unclear whether the observed neuronal modulations are meaningful for subsequent behavioral adjustments. This needs to be addressed in future studies.

In sum, we examined proactive control and binding processes in the context of task switching in patients with TS and matched healthy controls. Behavioral performance and electrophysiological modulations of proactive control involved in the reconfiguration of the new task were unaltered in TS patients. Importantly, C-cluster N2 and P3 modulations reflecting task set-response binding were altered, supporting the recent view that the integration of perception and action is processed differently in patients TS and may relate to the core symptoms of the disorder, sensory urges and motor tics. Future studies may further investigate the potential influence of different task characteristics and top-down processes such as proactive control on behavioral and neurophysiological binding processes in patients with TS.

## Synthesis

Reviewing Editor: Christopher Colwell, UCLA School of Medicine Decisions are customarily a result of the Reviewing Editor and the peer reviewers coming together and discussing their recommendations until a consensus is reached. When revisions are invited, a fact-based synthesis statement explaining their decision and outlining what is needed to prepare a revision will be listed below. The following reviewer(s) agreed to reveal their identity: Simon Morand-Beaulieu, Christian Beste. Note: If this manuscript was transferred from JNeurosci and a decision was made to accept the manuscript without peer review, a brief statement to this effect will instead be what is listed below.

The authors present an interesting study on cue-based task switching in N=25 patients with GTS including an analysis of neurophysiological data in comparison to a healthy control sample. The study is framed in a recent perception-action integration account of GTS. The authors find no evidence of altered cue-based task switching in GTS.

Both reviewers think that this study is well-conducted and clearly written. Both reviewers had specific comments designed to improve the ms and feel that the work will ultimately make an interesting contribution to the literature.

Reviewer 1

1. The sample size is small. While we appreciate the difficulty of recruiting TS participants in EEG research, the authors should clearly acknowledge the small sample size as a limitation.

2. The clinical assessment should be described in the methods section. If one skips Table 1 (I did because it’s at the end of the manuscript), they only learn about assessment of TS and associated symptoms in the “statistical analysis” section. Also, questionnaires should be written in long in the text before acronyms are used, as some readers may not be familiar with those. Finally, it should be indicated how these symptoms were assessed. Was it self-report or clinician-report?

3. Lines 221-231: I’m not very familiar with Bayesian statistics, but I wonder if these results should not be reported as BF10.

4. The readers interested in ERPs in may would prefer to be able to see the actual waveforms for TS participants and controls.

5. The section discussing task effects (lines 294-338) was a bit long. I think shortening this section could be beneficial.

Reviewer 2

1. At present, the framing of an altered perception-action integration in GTS is based on studies in patients do not have a chance to prepare for possible response set reconfigurations. While I see the point that using a cued switching paradigm to extend/test the idea of altered perception-action integration in GTS, it is, as it stands not well enough motivated. What is the precise rationale behind choosing a cued task switching paradigm which may pose the problem that subtle increase in perception-action rebinding/reconfiguration costs may not be evident?

2. Based on the behavioral and EEG data analysis the authors found no evidence for increased perception-action binding in GTS. However, especially for the EEG data this not unexpected given the methods. Also, previous work (e.g. Kleimaker et al., 2020, Brain) did not revealed evidence for increased perception action integration in GTS, when standard ERPs were analyzed. This was only possible when a temporal decomposition was performed. In fact, there is mounting evidence that perception-action integration (or event file) processes can only be analyzed once the EEG has been decomposed (cf. Verleger et al., 2014 Neuroimage, Takacs et al., 2020 HBM, Opitz et al., 2020 Neuroimage). The authors cite these studies, but they do not use the methods therein. The data analysis has to be repeated using this temporal decomposition. In fact, also during task switching effects are best visible once this temporal decomposition has been used (e.g. Wolff et al., 2017 BSAF). So, please do so to increase comparability of your findings.

3. I do see the point behind using the regression approach to analyze the data. However, this approach is not very common in the entire field of task switching and EEG. Therefore, I ask the authors to perform more traditional analyses. This will enable a better comparison of the results with other studies in the task switching and the GTS field. So, please do so to increase comparability of your findings. Specifically, authors used regressed single-trial EEG activity at each electrode and time point against task predictors, which were: Task Transition’, ‘Response Transition’ (coded as 0 = repeated response, 1 = switched response), a ‘Task Transition x Response Transition’ interaction and ‘Trial number’.

Instead, the author should perform a mixed model ANOVA using “group” (i.e. Tourette vs. controls) as within-subject factor and “task transition” and “response transition” as within-subject factor. For that, they will have to pre-specify time window of interest (i.e. where they quantify the mean amplitude at specific electrode positions). The latter can well be derived from previous literature using task switching, GTS and RIDE in task switching and/or GTS (see work from my group). In their present analysis the factor “group” factor is not well captured. It is only indirectly captured by running the regression of each single subject. This is not standard in the field and hampers comparability/replicability. The mentioned analysis need to be done for the standard ERP analysis as well as for each derived RIDE-cluster after performing RIDE.

4. In my opinion, the authors should better justify the experimental manipulations in the task switching paradigm. Please provide clear connections to cognitive theory and/or research in GTS to justify the design of the task switching experiment (e.g., Why was the CTI varied? Etc.).

5. Related to the point above. It is true that perception-action binding (event file) processes can be examined using task switching experiments. However, this is mainly because of the structure of task switching experiments (cf. BRAC framework by Frings et al. 2020, TICS). In relation to this I am actually wondering about the quite long intervals between successive trials. If I got is right, the trials were separated by 2500ms after which a CTI of up 500ms was presented before the target stimulus possibly calling for a task and/or response switch was introduced. This, however, means that that there was quite some time between task switches which reduces demands on event file reconfiguration. This, together with the CTI (and the induction of only residual switch costs due to the cueing) will very cause that the task becomes quite easy to conduct. This also becomes clear from the error rates. Therefore, the task is very likely to easy to really see differential effects between groups (floor effect). This should be discussed.

6. Did the authors observe differences in patients being medicated (even though they were asked to stop their medication 24h before testing)? If not, this should be acknowledged as a possible limitation.
