# Supplementary material for: Electrophysiological Correlates of Proactive Control and Binding Processes during Task Switching in Tourette Syndrome
Source: eNeuro. 2023 Apr 7;10(4):ENEURO.0279-22.2023. doi: 10.1523/ENEURO.0279-22.2023 (PMC10088983; doi:10.1523/ENEURO.0279-22.2023)
Supplement: Extended Data Figure 3-1 — Cue-locked switch positivity ANOVA results for the S-cluster and ERP. Significant ANOVA effects were followed up by an ANCOVA with Medication as covariate. Asterisk denotes statistical significance. P(H0|D) = probability of the null hypothesis being true given the observed data. See Extended Data Figure 3-2 for the corresponding waveforms. Download Figure 3-1, DOC file. [file enu-eN-NWR-0279-22-s02.doc]

**Extended Data Figure 3-1:**

| **Switch positivity** | **ANOVA** | | | | **ANCOVA** | | | |
| --- | --- | --- | --- | --- | --- | --- | --- | --- |
|  | *F*_(1,47)_ | *p* | *ηp²* | *P(H_0_\|D)* | *F*_(1,46)_ | *p* | *ηp²* | *P(H_0_\|D)* |
| **S-cluster** | | | | | | | | |
| Task Transition | 7.53 | .009 * | .138 | .155 | 6.87 | .012 * | .130 | .188 |
| Task Transition x Group | .145 | .705 | .003 | .866 |  |  |  |  |
| Group | .06 | .810 | .001 | .872 |  |  |  |  |
| **ERP** | | | | | | | | |
| Task Transition | 31.45 | < .001 * | .401 | .000 | 27.46 | < .001 * | .374 | .000 |
| Task Transition x Group | .65 | .425 | .014 | .834 |  |  |  |  |
| Group | .01 | .924 | .000 | .874 |  |  |  |  |
