# Supplementary figures and images for: Electrophysiological Correlates of Proactive Control and Binding Processes during Task Switching in Tourette Syndrome
Source: eNeuro. 2023 Apr 7;10(4):ENEURO.0279-22.2023. doi: 10.1523/ENEURO.0279-22.2023 (PMC10088983; doi:10.1523/ENEURO.0279-22.2023)

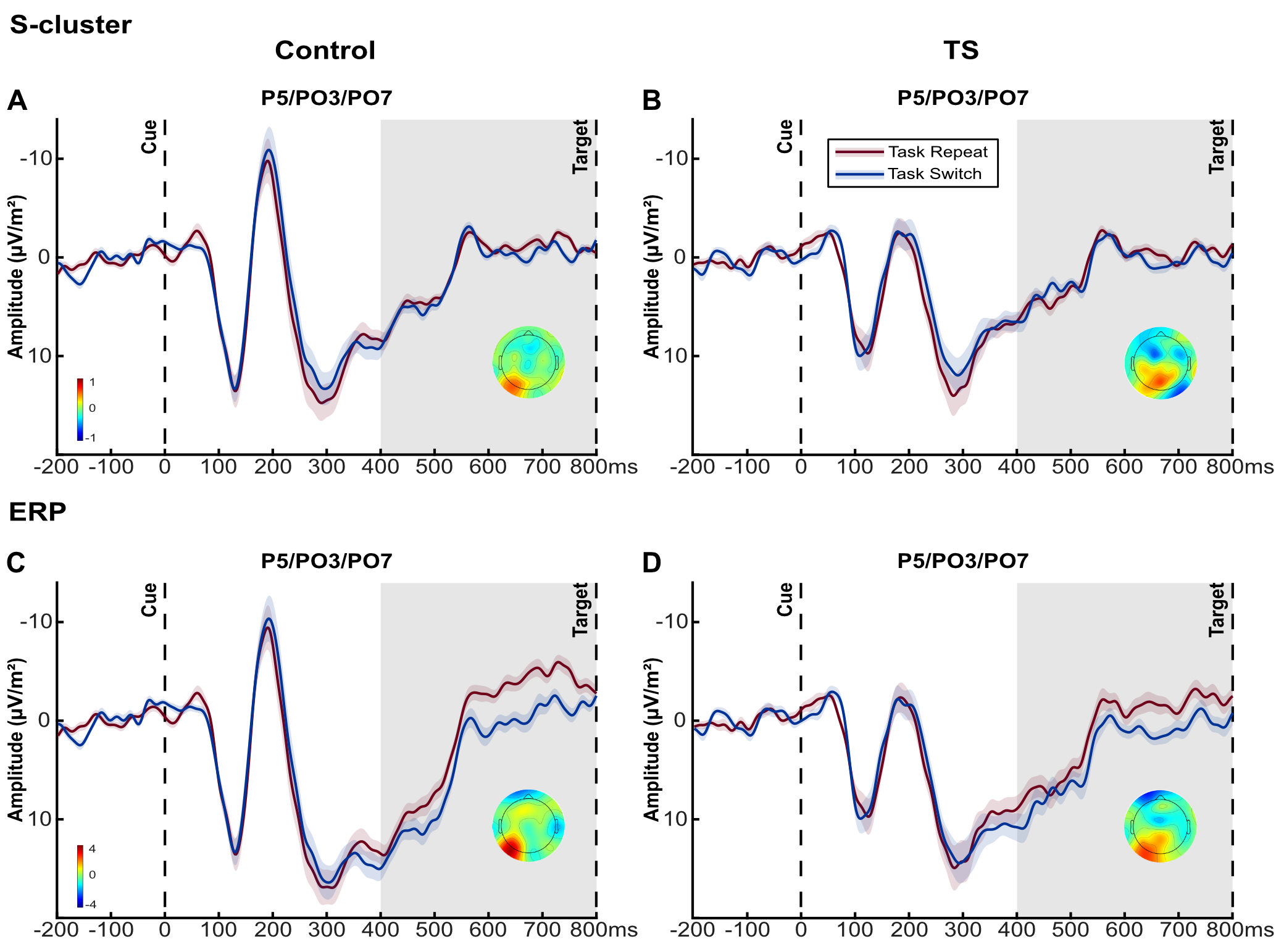

Supplement: Extended Data Figure 3-2 — Cue-locked switch positivity results. Grand average cue-locked waveforms at electrodes P5/PO3/PO7 in the S-cluster and standard ERP separately for controls and patients. A, S-cluster waveform in the control group. B, S-cluster waveform in the TS group. C, ERP waveform in the control group. D, ERP waveform in the TS group. Shading represents SE. The grey bar indicates the time window for mean amplitude quantification (400–800 ms). Scalp topography maps show the differences in mean amplitude (task switch–task repeat) in the respective time window. See Extended Data Figure 3-1 for the corresponding ANOVA results. Download Figure 3-2, TIF file. [file enu-eN-NWR-0279-22-s05.tif]

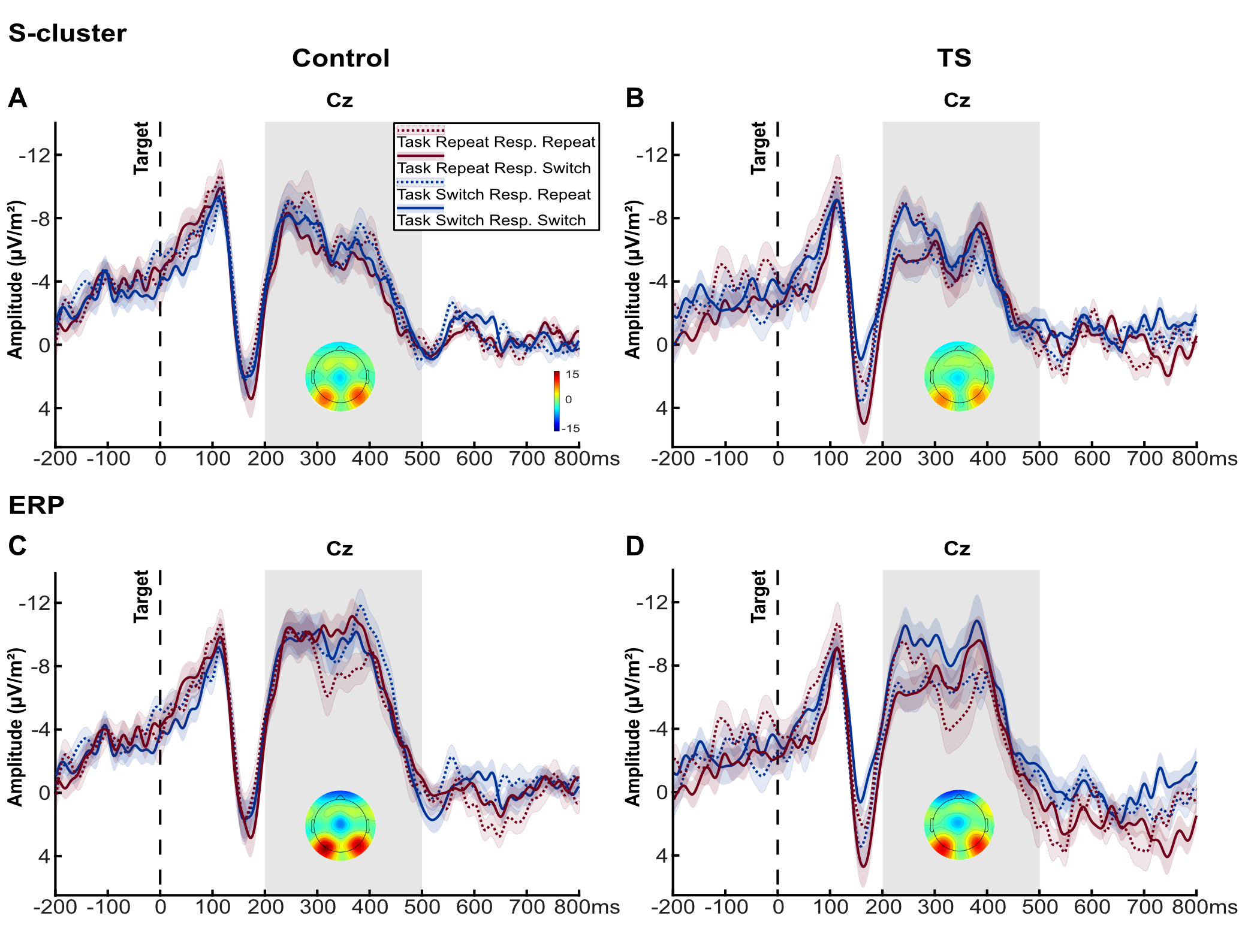

Supplement: Extended Data Figure 4-2 — Target-locked N2 results. Grand average target-locked waveforms at electrode Cz in the S-cluster and standard ERP separately for controls and patients. A, S-cluster waveform in the control group. B, S-cluster waveform in the TS group. C, ERP waveform in the control group. D, ERP waveform in the TS group. Shading represents SE. The grey bar indicates the time window for mean amplitude quantification (200−500 ms). Scalp topography maps show mean amplitudes in the respective time window. Resp. = Response. See Extended Data Figure 4-1 for the corresponding ANOVA results. Download Figure 4-2, TIF file. [file enu-eN-NWR-0279-22-s06.tif]

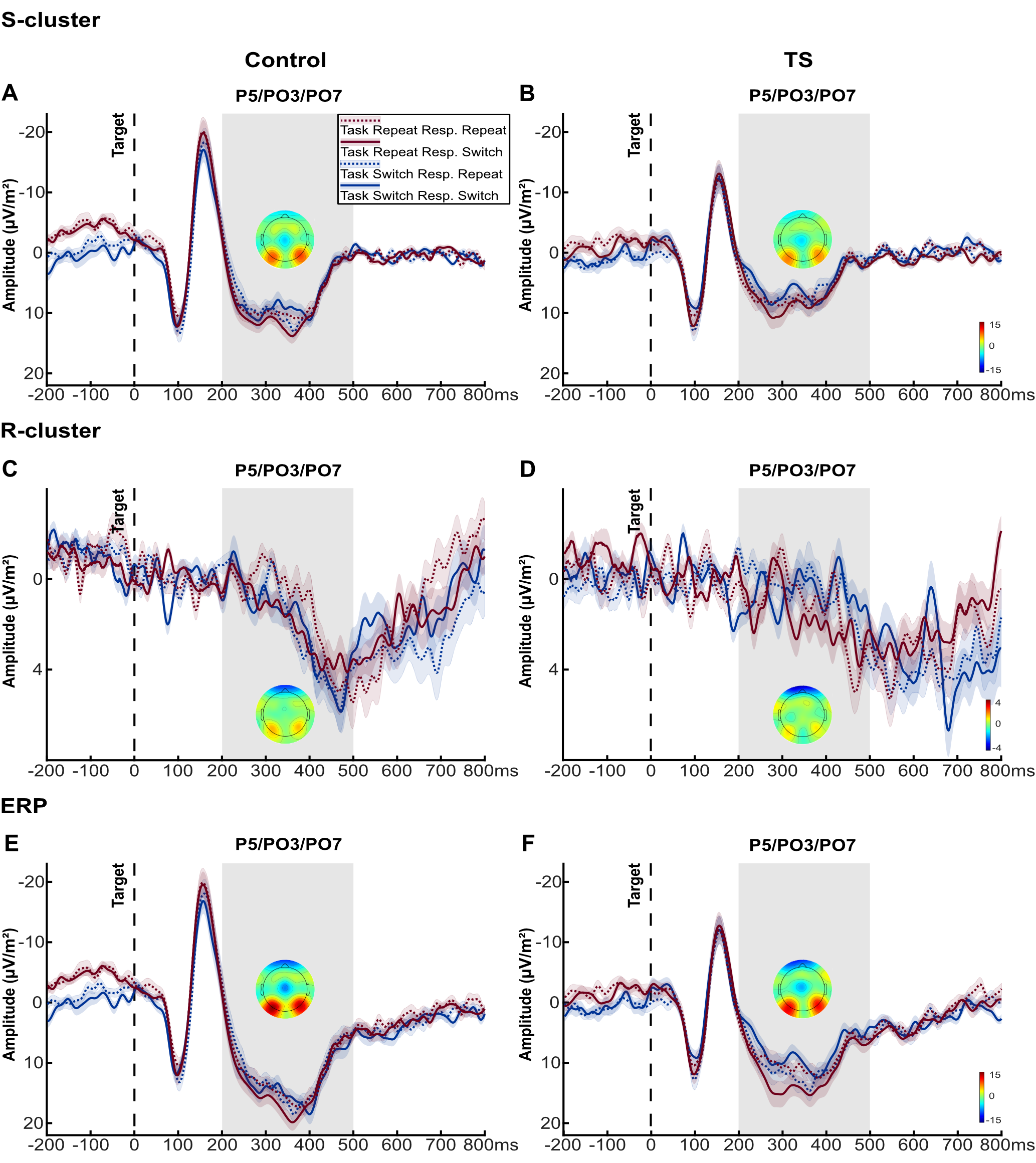

Supplement: Extended Data Figure 5-2 — Target-locked P3 results. Grand average target-locked waveforms at electrodes P5/PO3/PO7 in the S-cluster, R-cluster, and standard ERP separately for controls and patients. A, S-cluster waveform in the control group. B, S-cluster waveform in the TS group. C, R-cluster waveform in the control group. D, R-cluster waveform in the TS group. E, ERP waveform in the control group. F, ERP waveform in the TS group. Shading represents SE. The grey bar indicates the time window for mean amplitude quantification (200−500 ms). Scalp topography maps show mean amplitudes in the respective time window. Resp. = Response. See Extended Data Figure 5-1 for the corresponding ANOVA results for the R-cluster and ERP. Download Figure 5-2, TIF file. [file enu-eN-NWR-0279-22-s07.tif]
