# Supplementary material for: Electrophysiological Correlates of Proactive Control and Binding Processes during Task Switching in Tourette Syndrome
Source: eNeuro. 2023 Apr 7;10(4):ENEURO.0279-22.2023. doi: 10.1523/ENEURO.0279-22.2023 (PMC10088983; doi:10.1523/ENEURO.0279-22.2023)
Supplement: Extended Data Figure 4-1 — Target-locked N2 ANOVA results for the S-cluster and ERP. Significant ANOVA effects were followed up by an ANCOVA with Medication as covariate. Asterisk denotes statistical significance. P(H0|D) = probability of the null hypothesis being true given the observed data. See Extended Data Figure 4-2 for the corresponding waveforms. Download Figure 4-1, DOC file. [file enu-eN-NWR-0279-22-s03.doc]

**Extended Data Figure 4-1:**

| **N2** | **ANOVA** | | | | **ANCOVA** | | | |
| --- | --- | --- | --- | --- | --- | --- | --- | --- |
|  | *F*_(1,47)_ | *p* | *ηp²* | *P(H_0_\|D)* | *F*_(1,46)_ | *p* | *ηp²* | *P(H_0_\|D)* |
| **S-cluster** | | | | | | | | |
| Task Transition | .06 | .801 | .001 | .871 |  |  |  |  |
| Response Transition | .15 | .696 | .003 | .866 |  |  |  |  |
| Group | .26 | .616 | .005 | .860 |  |  |  |  |
| Task Transition x Group | .17 | .682 | .004 | .865 |  |  |  |  |
| Response Transition x Group | .89 | .351 | .019 | .816 |  |  |  |  |
| Task Transition x Response Transition | 5.07 | .029 * | .097 | .362 | 3.99 | .052 | .080 | .477 |
| Task Transition x Response Transition x Group | .03 | .873 | .001 | .874 |  |  |  |  |
| **ERP** | | | | | | | | |
| Task Transition | 2.59 | .114 | .052 | .652 |  |  |  |  |
| Response Transition | 2.33 | .133 | .047 | .681 |  |  |  |  |
| Group | 1.31 | .258 | .027 | .781 |  |  |  |  |
| Task Transition x Group | .62 | .434 | .013 | .835 |  |  |  |  |
| Response Transition x Group | .73 | .396 | .015 | .827 |  |  |  |  |
| Task Transition x Response Transition | .00 | .973 | .000 | .875 |  |  |  |  |
| Task Transition x Response Transition x Group | 3.54 | .066 | .070 | .541 |  |  |  |  |
