# Supplementary material for: Electrophysiological Correlates of Proactive Control and Binding Processes during Task Switching in Tourette Syndrome
Source: eNeuro. 2023 Apr 7;10(4):ENEURO.0279-22.2023. doi: 10.1523/ENEURO.0279-22.2023 (PMC10088983; doi:10.1523/ENEURO.0279-22.2023)
Supplement: Extended Data Figure 5-1 — Target-locked P3 ANOVA results for the R-cluster and ERP. Significant ANOVA effects were followed up by an ANCOVA with Medication as covariate. Asterisk denotes statistical significance. P(H0|D) = probability of the null hypothesis being true given the observed data. See Extended Data Figure 5-2 for the corresponding waveforms. Download Figure 5-1, DOC file. [file enu-eN-NWR-0279-22-s04.doc]

**Extended Data Figure 5-1:**

| **P3** | **ANOVA** | | | | **ANCOVA** | | | |
| --- | --- | --- | --- | --- | --- | --- | --- | --- |
|  | *F*_(1,47)_ | *p* |  | *P(H_0_\|D)* | *F*_(1,46)_ | *p* | *ηp²* | *P(H_0_\|D)* |
| **R-cluster** | | | | | | | | |
| Task Transition | .32 | .573 | .007 | .856 |  |  |  |  |
| Response Transition | 1.12 | .295 | .023 | .797 |  |  |  |  |
| Group | 1.71 | .198 | .035 | .745 |  |  |  |  |
| Task Transition x Group | 3.66 | .062 | .072 | .527 |  |  |  |  |
| Response Transition x Group | .01 | .925 | .000 | .874 |  |  |  |  |
| Task Transition x Response Transition | .436 | .512 | .009 | .848 |  |  |  |  |
| Task Transition x Response Transition x Group | .032 | .860 | .001 | .873 |  |  |  |  |
| **ERP** | | | | | | | | |
| Task Transition | 6.28 | .016 * | .118 | .245 | 8.59 | .005 * | .157 | .095 |
| Response Transition | 2.92 | .094 | .058 | .615 |  |  |  |  |
| Group | 1.24 | .272 | .026 | .788 |  |  |  |  |
| Task Transition x Group | 1.27 | .265 | .026 | .784 |  |  |  |  |
| Response Transition x Group | 2.02 | .162 | .041 | .714 |  |  |  |  |
| Task Transition x Response Transition | 2.79 | .101 | .056 | .630 |  |  |  |  |
| Task Transition x Response Transition x Group | 2.47 | .123 | .050 | .666 |  |  |  |  |
